# Assessment of hematological adverse drug reactions to antiretroviral therapy in HIV positive patients at Kasturba Hospital Manipal

**DOI:** 10.1186/1471-2334-12-S1-P55

**Published:** 2012-05-04

**Authors:** Poornima Pulagam, Radhakrishnan Rajesh, Sudha Vidyasagar, Danturulu Muralidhar Varma

**Affiliations:** 1 Department of Pharmacy Practice, Manipal College of Pharmaceutical Sciences, Manipal University, Manipal – 576 104, Karnataka, India; 2 Department of Medicine, Kasturba Medical College, Manipal University, Manipal – 576 104, Karnataka, India

## Background

HIV-infected patients have a higher risk of developing hematological adverse drug reactions (ADRs) than the general population and have a significant impact on patients’ current and future care options. The study was to determine the causality, the incidence rate, severity pattern of occurrence of hematological ADRs associated with highly active antiretroviral therapy in HIV positive patients.

## Methods

Prospective observational study conducted at medicine department, Kasturba Hospital over a period of 6 months. Enrolled HIV positive patients were intensively monitored for hematological ADRs associated with fixed dose of highly active antiretroviral therapy. Causality of adverse drug reactions was assessed by using WHO probability scale and also with Naranjo's algorithm.

## Results

Monitoring of 70 HIV positive patients (58 males and 12 females) with fixed dose drug combinations of antiretroviral therapy by active pharmacovigilance identified 47.3% cases of anemia, 15.7% cases of leucopenia, 21% cases of pancytopenia, 5.2% of eosinophilia, 10.5% cases of bicytopenia. The incidence rate of hematological adverse drug reactions was 37.9% and most of the reported ADR’s were definitely predictable and preventable. Fifty percent of these ADR’s were lead to hospitalization and seventy percent of these ADR’s were of moderate in severity. Hematological ADRs were highest with Zidovudine + Lamivudine + Nevirapine (68.4%) and Zidovudine + Lamivudine + Efavirenz (31.57%) combinations.

## Conclusion

With the increasing access to use of highly active antiretroviral in India, clinician must focus for routine monitoring of hematological investigations for early detection and prevention of adverse effects associated with highly active antiretroviral therapy.

